# Polymer-Like Self-Assembled Structures from Particles with Isotropic Interactions: Dependence upon the Range of the Attraction

**DOI:** 10.1021/acs.langmuir.1c00719

**Published:** 2021-05-05

**Authors:** Sara Haddadi, Hongduo Lu, Marcus Bäcklund, Clifford E. Woodward, Jan Forsman

**Affiliations:** † Theoretical Chemistry, Lund University, P.O. Box 124, S-22100 Lund, Sweden; ‡ University College, University of New South Wales, ADFA, Canberra, Australian Capital Territory 2600, Australia

## Abstract

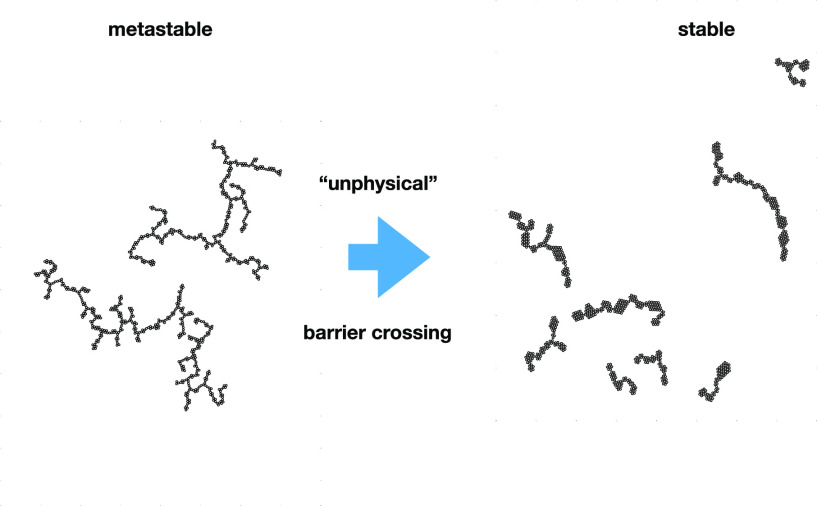

We conduct Metropolis Monte Carlo simulations on models of dilute colloidal dispersions, where the particles interact via isotropic potentials of mean force (PMFs) that display a long-ranged repulsion, combined with a short-ranged and narrow attraction. Such systems are known to form anisotropic clusters. There are two main conclusions from this work. First, we demonstrate that the width of the attractive region has a significant impact on the type of structures that are formed. A narrow attractive well tends to produce clusters in which particles possess fewer neighbors than in systems where the attraction is wider. Second, metastable clusters appear to persist in the absence of specific simulation moves designed to overcome large energy barriers to particle accumulation. The so-called “Aggregation-Volume Bias Monte Carlo” moves were previously developed by Chen and Siepmann, and they facilitate particle exchanges between clusters via unphysical moves that bypass high energy intermediate states. These facilitate the progression of metastable clusters to equilibrium clusters. Metastable clusters are generally large with significant branching of thin filaments of aggregated particles, while stable clusters have thicker backbones and tend to be more compact with significantly fewer particles. This general behavior is observed in both two- and three-dimensional systems. In two dimensions, less anisotropic clusters with backbones possessing lattice structures will occur, particularly for systems where the particles interact with a PMF that has a relatively wide attractive region. We compare our results with PMF calculations established from a more specific model, namely weakly charged polystyrene particles, which carry a thin surface layer of grafted polyethylene oxide polymers in aqueous solution. We hope that our investigations can serve as crude guidelines for experimental research, aiming to construct linear or branched polymers in aqueous solution built up by colloidal monomers that are large enough to be studied by confocal microscopy. We suggest that metastable clusters are more relevant to experimental scenarios where the energetic barriers are too large to be surmounted over typical timescales.

## Introduction

Understanding self-assembly of colloidal particles is paramount to the successful synthesis of supramolecular structures with desirable properties.^2−10^ Significant progress has been made in recent years on the synthesis of colloidal particles that interact via anisotropic interactions^11−13^ in a manner that is analogous to the asymmetry usually seen in interactions between protein molecules. These include the so-called “Janus”^12^ and “patchy” particles.^13^ The presence of short-ranged anisotropic interactions may naturally cause colloidal particles to self-assemble into highly asymmetric structures, which can be advantageous for various technical applications. One drawback with such a strategy, however, is the technical challenge of synthesizing particles with the requisite structural asymmetry.

While a dispersion of colloidal particles with spherically symmetric (isotropic) interactions may form fluid phases, which are (on average) spatially uniform, theoretical, and experimental work, has demonstrated that such phases may contain clusters, which are highly asymmetric. This has been observed, for example, when the particles interact via a short-ranged attraction and a long-ranged repulsive potential. This is not limited to heteroaggregation, but can occur in dispersions containing identical particles. In some cases, these clusters are dynamically favored and, once formed, must negotiate nearly insurmountable free energy barriers in order to attain thermodynamically more stable flocculated phases. The tendency for these systems to form gels via percolation has also been demonstrated.

An early work on these kinds of dispersions includes geometrical analyses by Thomas and McCorkle^14^ and by Sonntag and co-workers.^15^ In both these cases, the focus was on flocculating particles interacting via the standard DLVO^16,17^ potentials of mean force (PMFs). The DLVO potential includes a short-ranged Hamaker attraction and long-ranged electrostatic repulsion. This early work was complemented by further theoretical progress^18^ and by other experimental contributions,^19^ as summarized by Dukhin et al..^20^ More recently, Sciortino, Zaccarelli, and co-workers^21,22^ have performed dynamic simulations of dispersions, where strongly anisotropic structures arise, composed of colloidal particles whose PMFs possess similar characteristics. They used a generalized Lennard-Jones potential, as suggested by Vliegenhart and Lekkerkerker,^23^ to model the short-ranged attractive component of the interaction. The aggregates that formed were highly branched, with a “thick” backbone, that showed some resemblance to the so-called Bernal spirals.^24^ Mani et al.^25^ found similar structures in a simulation study on equilibrium clusters. However, they concluded that for their systems, the Bernal spirals were only metastable. Also, of note is a reasonably recent experimental study by van Schooneveld et al.,^26^ who observed highly anisotropic structures for particles interacting via a long-ranged electrostatic repulsion and a short-ranged depletion attraction. The particles were quite large (∼ μm) and could thus be detected by confocal microscopy. They used a nonaqueous solvent, which facilitated an extremely low ionic strength giving a repulsive range similar to the particle size.

The picture that is obtained from these theoretical and experimental studies is that the magnitude and range of the long-ranged repulsive and short-ranged attractive contributions to the potential play a significant part in determining the stability and structure of the emergent clusters and their ability to flocculate and form condensed phases or intervening gels. For example, it has been suggested that PMFs with strong repulsions can give rise to ground-state (minimum energy) clusters of either finite or infinite size. These may of course not survive the presence of thermal energy and the resulting entropic contributions to the free energy will favor the breaking up of these ground-state structures. Nevertheless, the presence of spherical or columnar phases (as clusters or holes) is indicated at high volume fraction when the repulsive interaction overcomes the increased attraction that is created by “bonding” to many neighbors.^27^ If instead the short-ranged interactions dominate the repulsions, the thermodynamically favored ordered structure will be a crystalline solid, as illustrated by Mani et al.^25^

In this study, rather than focusing on condensed phases or the approach to them, we will explore how the range and magnitude of the attractive interactions (relative to the repulsions) affect the cluster morphology at low volume fractions, especially when large free energy barriers to aggregation are present ∼20 *k*_B_*T* (where *k*_B_*T* is the thermal energy). Previous theoretical studies have tended to focus on free energy barriers (created by weaker repulsions) an order of magnitude smaller than those which we will study here.^25^ The PMFs that we will explore in this work do have a significant repulsion and give rise to ground-state structures, which form the so-called Bernal spirals in three dimensions^24^ and double columnar clusters in two dimensions. It is in this parameter regime that we investigate the importance of the range of the short-ranged contribution to the PMF, in determining cluster morphology at finite temperatures. Such PMFs have already been encountered in an earlier theoretical study^28^ and were used to explain experiments on weakly charged polystyrene (PS) particles with thin grafted layers of poly-ethylene oxide (PEO).^4^ The theory was able to successfully describe anomalous temperature dependence in the gelation of the PS particles, using model PMFs consisting of a short-ranged attraction due to bridging of the grafted PEO chains and a long-ranged electrostatic repulsion, giving rise to barriers ∼20 *k*_B_*T*.

A fluid model incorporating a pair potential with a deep and narrow minimum, combined with a substantial free energy barrier and a long-ranged repulsive tail, will be computationally challenging to simulate. Most previous theoretical efforts in this field have utilized dynamic simulation methods, a notable exception being a rather recent work by Sweatman et al.^29^ The present investigation relies on Metropolis Monte Carlo (MC) simulations (in the Canonical Ensemble) starting from random particle distributions. There are advantages and disadvantages with either simulation protocol. With dynamical methods, one is able to study time-dependent properties, but when strong free energy barriers are present, the approach to equilibrium can be prohibitively slow. This is seen in experiments. For example, van Schooneveld et al.^26^ still observed structural changes in colloidal dispersions (with these types of interactions) after several weeks. The advantage of MC methods is that we are able to implement essentially unphysical moves to effectively traverse large energetic barriers to more quickly arrive at equilibrium distributions. However, one should remain cautious when comparisons are made with experiments, as it is possible that certain moves may penetrate free energy barriers that would in essence be insurmountable in the timescale of real experiments. We will demonstrate that, at least in three-dimensional (3D) systems, there may exist metastable cluster phases with geometries and typical sizes, which can be considerably different from those of the properly equilibrated system. The transition from the former to the latter can only be realized via very efficient, but quite “unphysical” MC moves, and we argue that in an experimental scenario, the system is likely to remain in the metastable state. These observations appear to be corroborated by recent experimental findings.^30^

In the Supporting Information, we include MC simulations of one of the systems that were studied by Sciortino et al.^21^ (where barrier heights and minima were about an order of magnitude weaker than in the systems studied here). We obtained similar structures and interaction energies as in those reports. Our analyses are also complemented by those in which the particles are constrained to a two-dimensional (2D) surface. A 2D system is more easily visualized by configurational snapshots and can model experimental systems where the particles adsorb to a surface or at an interface between two immiscible liquids.

In summary, the present work differentiates itself from previous studies in this field in the following ways:

- The free energy barriers are about an order of magnitude stronger than those that have been commonly employed in previous work. We argue that such high barriers are often encountered in experimental scenarios.
- Our work highlights the impact that the width of the attractive potential regime has on the structures of the clusters formed.
- We study and compare results from 2D and 3D systems. The former are not only interesting in their own right, but have direct relevance to experimental analyses, such as confocal microscopy.
- We compare our PMFs to those established by more detailed theoretical models with explicit account of temperature-responsive polymers grafted onto the particle surfaces. These models have in turn been previously validated against experimental data.^4,28^ We argue that this makes our work potentially useful as a guide for experimental efforts to produce these types of structures in aqueous solution.
- We demonstrate the occurrence of metastable clusters, which tend to be extensively branched, with a rather narrow backbone. Here, very large free energy barriers prevent further progression to fully equilibrated clusters, unless somewhat unphysical, but highly efficient, “Aggregation-Volume Bias MC” moves are implemented.

## Models and Methods

As stated in the Introduction, the model PMFs we use in this work were inspired by earlier experimental and theoretical work on aqueous dispersions of PS particles with grafted PEO polymers.^4,28^ The model consists of a monodisperse dispersion of hard spheres with a diameter, σ = 2800 Å. The grafted PEO chains were quite short (∼45 mers) and mediate an attraction at elevated temperatures through bridging between particles. In addition, the particles possess a weak charge, giving rise to a long-ranged repulsion at low ionic strength. The model we propose here will not explicitly invoke the complex classical polymer density functional theory (DFT) approach adopted in the earlier work.^28^ Instead we approximate the qualitative and quantitative features of the PMF using the following analytical expression (Morse potential), where *S* is the separation between the surfaces of the particles
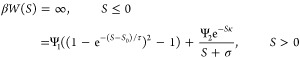
1as mentioned above, β = 1/*kT* is the inverse thermal energy. The Morse potential parameter *S*_0_ = 10 Å ensures a relatively short-ranged minimum compared to σ. In this work, we will explore structural changes to the particle cluster morphology as a response to the width of the attractive well in the PMF, which can be tuned by the τ parameter. The depth of the attraction remains unchanged upon varying τ. We set a “reference value” for a Morse amplitude factor of Ψ_1_ = 70, but we will also consider a slightly reduced value (Ψ_1_ = 60) in one case.

The range of the (screened Coulomb) repulsion is given by κ^–1^, the Debye screening length, which is determined by the ionic strength, the dielectric constant of the solvent, and the temperature. The prefactor, Ψ_2_, can be explicitly related to the particle valency, *Z*, using
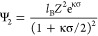
2where *l*_B_ is the Bjerrum length. As we hope that our results can serve as a guide for experimental investigations in aqueous dispersions, we have chosen practically realizable values for the valency, *Z*, and the ionic strength. Guided by the PS + grafted PEO model system, we will then assume a temperature of 373 K and a relative dielectric constant of 55 (close to theta temperature for PEO and the corresponding dielectric constant for water). This implies a Bjerrum length of about 8 Å. In all the cases we investigate, we use *Z* = 176 and κ = 0.0007 Å^–1^, which means that the Debye length is slightly shorter than the particle size.

The parameter values chosen were guided to some extent by previous experimental and theoretical results.^4,28^ For example, we match typical attractive well depths of the PS + grafted PEO model system, as we shall show below. Moreover, the particle valencies and Debye lengths correspond to an expected weak surface charge density in an aqueous dispersion at low ionic strength.^4,28^ Thus, the system we describe is experimentally realizable and the particles are large enough to allow for analysis by confocal microscopy. As stated above, τ, that is, the range of the attraction was varied in our study. This essentially corresponds to varying the length of the grafted PEO chains.

**Figure 1:**
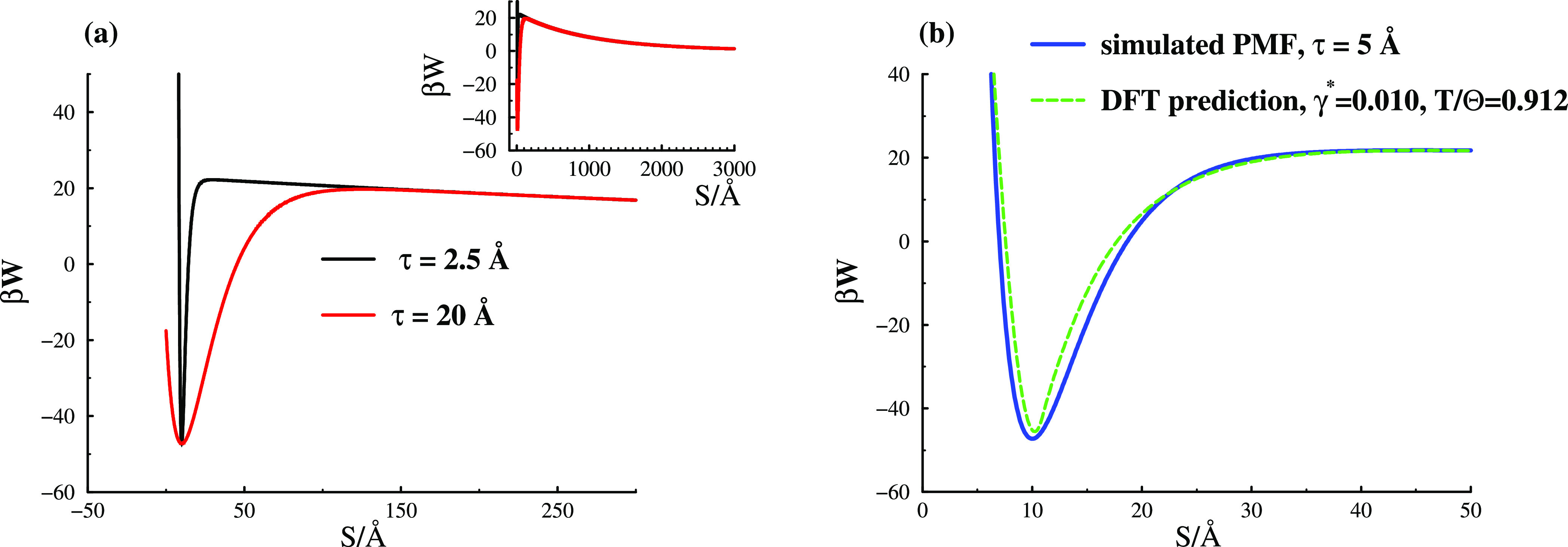
Potential of mean forces. (a) Examples, with various widths of the attractive minimum. (b) Demonstration that a narrow PMF can be generated by statistical-mechanical (classical) DFT calculations of a specific model with an explicit representation of temperature-responsive grafted polymers, which in turn has been validated against experiments.^4,28^ Wide attractions could not be reproduced by this model, but we anticipate that depletion interactions would be a viable option to generate such PMFs.

In order to vary the width (but not the depth) of the attractive region of the PMF region, we will consider four different values for the decay length of the short-ranged Morse potential τ = 2.5, 5, 20, and 40 Å. The short-ranged regime of two selected PMFs is highlighted in the main graph of Figure 1a. We note very substantial free energy barriers of around 20 *kT*. The variation in the range of the attractive regime is barely discernible, when viewed at a scale that captures the long-ranged tail (inset). Nevertheless, magnifying that region (shown in the main plot), we note that the width of the attraction, as measured by the position of the barrier maximum, ranges over about an order of magnitude.

In Figure 1b, we show the result of replacing the Morse potential (the attractive component in eq 1) by predictions from classical polymer DFT calculations using the more complex explicit model of a grafted PEO layer (45 mers) on PS spheres in an aqueous solution. To calculate the latter, we used a DFT approach as described by Xie et al.,^28^ which the reader should refer to for details. It is instructive to point out here though that this theoretical model successfully reproduced the gelation behavior observed in experiments,^4^ including the dependence on temperature and grafting density.

From our fitting attempts, we see that a narrow analytic PMF (eq 1), can be reasonably well captured by the explicit model. For the latter we used a (reduced) polymer grafting density of about γ* = γ*d*^2^ ∼ 0.01, where γ is the grafted end-monomer density per unit area on the particle surface and *d* is the monomer diameter. This grafting density is of the same order as that which can be inferred from the experimental systems.^4^ Those experiments used a PEO to PS weight ratio of between 0.06 and 0.24, with an average PEO length of approximately 45 monomers. The average PS particle radius was approximately 1400 Å. Assuming that all PEO chains were grafted gave a reduced grafting density of γ* ≈ 0.0086 for the “L6” system. In order to arrive at a wide attraction (such as with τ = 20 or 40 Å), one would have to utilize much longer grafted chains or produce the attraction by using depletion forces instead.

Dispersion attractions have not been included in these models, but these typically give quite weak contributions to the overall PMF in these systems.^28^ It should also be noted that the approximations underlying the assumption of a screened Coulomb repulsion will most likely break down at very short particle separations, where the discrete nature of charges and solvents plays a role. Fortunately, these modeling problems arise in the regime (below a nanometer or so), where the interaction is dominated by the very strong short-ranged part, suggesting that the effects of such “perturbations” are likely to have a rather moderate influence. Furthermore, because the particles are very weakly charged with monovalent dissolved ions, we expect the linearized Poisson–Boltzmann to be quite accurate at a long range.

### Energy-Minimized Structures

As noted earlier, energy minimization in three dimensions will generate Bernal spirals^24^ for all our investigated PMFs. The Bernal spiral has the structure of a close-packed arrangement of spheres in a cylinder. Each sphere has six nearest neighbors, taking good advantage of the short-range attractive interactions. Furthermore, of all other cylindrical packings of spheres with this number of nearest neighbors, the Bernal spiral has the smallest cylindrical radius, thus reducing the associated electrostatic repulsions. As giving the system a finite temperature will only increase the entropy, we do not expect that our systems will display an extended 3D crystalline phase. More likely, its ordered phases may consist of spherical, columnar, or lamellar clusters.^27^

**Figure 2:**
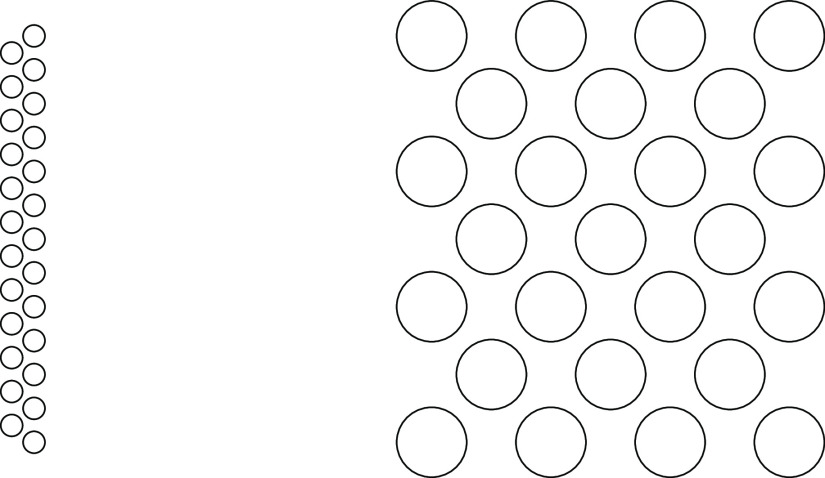
Illustration of two possible energy-minimized structures in two dimensions (see main text). Left: a “double rod”. Right: an extended 2D lattice.

The situation is more subtle in two dimensions. The energy difference between rods, consisting of two staggered rows, and an extended triangular (2D) lattice is typically small for the PMFs we used here. These structures are depicted in Figure 2. Specifically, the extended 2D lattice leads to an adhesive interaction energy per particle that is a few percent stronger than the rodlike structure due essentially to the positive line energy of the rods, which is not compensated by the lower electrostatic repulsion. However, increasing the Debye length, to about 2500 Å, is enough to make the rod more stable than the triangular lattice. The close similarity in energy for the rod and the lattice will impact the finite temperature structures, especially (as it turns out) for PMFs with a broad minimum, as we will discuss below.

### Simulation Details

All 3D and 2D simulations were performed using 800 particles (*N* = 800). In three dimensions, these were distributed within a cubic volume with a side length of 195,347 Å, giving a volume fraction of approximately 0.12%. In our 2D simulations, the particles were distributed on a square with a side length of 390,694 Å. The use of such a low concentration prevents the formation of extended aggregates (e.g., gels), thereby allowing us to study the structure of isolated clusters, which form in what is essentially a pseudo-gas phase of the effective particle fluid. In experimental scenarios that aim to isolate supramolecular structures formed from colloidal particles, this would also be the preferred concentration regime. Attractive and repulsive interactions between particles were truncated at 15 decay lengths. That is, the PMFs were truncated at 15 τ and 15/κ for the attractive and repulsive parts, respectively. This was large enough to preclude any significant effects due to truncating the PMF. Square (2D) or cubic (3D) periodic boundary conditions were applied for all simulated systems.

In an attempt to circumvent major convergence problems in the simulations, we utilized a number of different types of (attempted) particle moves:

1 Random single particle displacements within a cube of side length 2δ (centered on the particle coordinate) with δ just a few Å. This provides efficient sampling of particle position optimizations close to an energy minimum (akin to vibrations in a dynamic system). In this context, we note that the top of the repulsive barriers in the PMFs occurs at particle surface separations, ranging from about 30 Å to about 220 Å, for the different widths (τ) investigated.
2 Random single particle displacements, within a larger cube of side length 2Δ, centered on the particle coordinate, with Δ typically being about 1–2 particle diameters. These moves will allow particles find new low energy positions within a cluster.
3 The attempted single particle moves to a randomly chosen position within the simulation box. This type of move will allow isolated particles to enter an existing cluster without climbing the large barrier and, in principle, also allow for exchange of particles between clusters. However, the latter process will in practice almost never occur.
4 AVBMC moves, developed by Chen and Siepmann.^1^ These facilitate “unphysical”, but highly efficient exchange of particles between existing clusters. As we shall see, this type of move will allow for the transformation of metastable conditions to fully equilibrated systems. Chen and Siepmann subsequently developed alternative formulations,^31^ and we have also tested the “AVBMC2” version, but because we were unable to detect any significant increase of simulation efficiency, we have mostly adhered to the initial (“AVBMC1”) version.
5 Standard simple cluster moves, using a continuum space generalization of the moves suggested by Wu et al.^32^ We utilized a spherical cluster, with a radius that is randomly chosen between σ and some maximum value *R*_max_, where *R*_max_ is chosen to capture the largest clusters, as estimated from radial distribution functions sampled in a previous simulation. Two particles are deemed to belong to the same cluster if their (nearest) surface separation is less than 100 Å (this distance criterion was varied across a large range of values with identical results). According to the standard (simplest) cluster move criterion, any cluster move that leads to a change of the number of members in the cluster, will be rejected, in order to ensure microscopic reversibility.

In all cases, more than 2 × 10^11^ attempted configurations were generated. It should be noted that if we were to use only Type 1 moves (listed above), then no clusters would form within the simulation lengths used, nor would any be likely to form, even for much longer runs. This implies that dynamical simulation methods are unlikely to generate clusters of any substantial size within a manageable simulation time. This is in line with experimental observations, where structural equilibration often takes several weeks.^26,30^

The moves of Types 3 and 4 involve long-ranged displacement of particles and allow some isolated particles to adopt “bonded” positions within clusters, while bypassing repulsive energy barriers and for this reason may be considered somewhat “unphysical” compared with dynamically determined moves. This notwithstanding, they can also be interpreted as means by which kinetic barriers, which play a minimal role in determining equilibrium distributions, can be efficiently bypassed. Type 3 moves, are purely random long-ranged moves and by themselves are able to create clusters of significant size and branched morphology. These reflect rare chance encounters between particles that are able to find bonded configurations. Once a particle becomes part of a cluster, though it has little likelihood of leaving it. Moves of Type 2 will allow thus formed clusters to equilibrate their structures via smaller random displacements. We suggest that simulations that include Type 3 moves (and not Type 4 moves) probably best represent experimental scenarios, wherein metastable (kinetically constrained) clusters occur.

Type 4 (AVBMC) moves are specifically designed to sample cluster configurations by identifying the bonding regions around randomly selected particles and trialling moves into those spaces. Furthermore, particles are also more efficiently able to leave clusters. Bond formation is energetically driven, and bond breaking is driven by entropic considerations. The latter therefore generally requires longer simulation times in order to be properly sampled, unless more efficient simulation moves are introduced, such as the various AVBMC methods. For this reason, simulations that include Type 4 moves are better able to predict true equilibrium configurations, though these may not be representative of what is observed in experiments of finite duration.

We conclude this section by stating that we cannot escape the qualitative nature of these timescale arguments, and clearly, the matching of MC moves to dynamical events can only be speculative. Over very long timescales, a hierarchy of kinetically constrained events will unfold in these systems, which will depend upon the initial state as well. We have assumed the latter is a random configuration of isolated colloids. Ultimately, the veracity of our assumptions can only be ascertained by comparisons with extremely long (probably impractically long) dynamical simulations or by experiments.

## Results and Discussion

Our aim in this work is to study the structural properties of the clusters, once the colloidal dispersion has become metastable or has reached complete equilibrium, which depends upon the type of MC moves included in the simulation (as discussed above). We do this by monitoring the instantaneous value for the interaction energy per particle, *U*/*N*, during the course of the simulation. This energy is due primarily to the large cohesive energy expected for particles bonded to form clusters. Values were taken at regular intervals and plotted against the number of attempted MC configurations. In all cases, the initial configuration consisted of a random distribution of particles within the simulated volume.

The average number of particles per cluster, that is, the “cluster size”, is a quantity of obvious interest. If *p*(*n*) is the probability that a randomly chosen cluster has *n* particles, then the average cluster size is
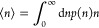
3

From *p*(*n*), one can obtain the integrated cluster size distribution, *P*_c_(*N*_c_), defined as the probability that a randomly selected cluster contains at most *N*_c_ monomers, that is,
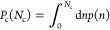
4

While *P*_c_(*N*_c_) is a measure of overall aggregation, it does not provide information concerning the shape or the bonding structure within the clusters. As a measure of the latter, we determined the probability *P*_b_(*k*) that a particle in a cluster is bonded (according to the cluster criterion) to at least *k* other particles. Finally, rather than using additional measures for the cluster shapes, we will simply show snapshots of typical converged configurations. The appearance of the clusters in these can then be correlated with the probability functions described above. In two dimensions, these snapshots simply amount to a plot of the *x*, *y* coordinates of the particles. For 3D plots, we have used the VMD software (developed with NIH support by the Theoretical and Computational Biophysics group at the Beckman Institute, University at Illinois at Urbana-Champaign).^33^ These structural data were collected from a single snapshot of an equilibrated system.

### 2D Systems

Here, we consider systems with a wide (τ = 40 Å) and a narrow (τ = 2.5 Å) attractive portion in the PMF and also investigate the role of including AVBMC moves.

**Figure 3:**
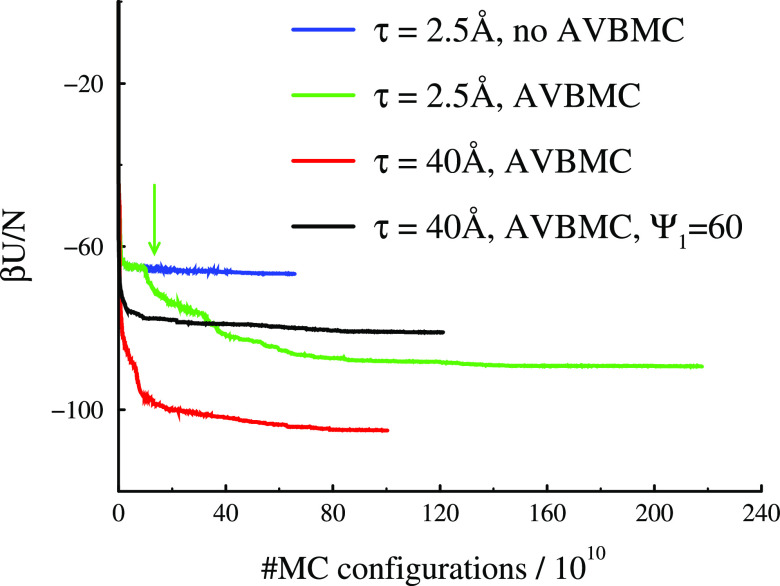
Energy convergences for the 2D systems.

In Figure 3, we show how the interaction energy per particle, *U*/*N*, varies with the number of attempted MC moves. In most cases, AVBMC moves are activated after an initial pre-equilibration. However, for the system with the tightest attractive potential, τ = 2.5 Å, the pre-equilibrated system was continued along two different paths, one of which was allowed to progress without AVBMC moves. The “split” into two distinct simulation paths is indicated by a green arrow in Figure 3. We note that, without the AVBMC moves, a plateau is reached, indicating that the system has likely adopted a metastable state. Switching on the AVBMC gives rise to a sudden lowering of *U*/*N*, until a lower plateau region is eventually reached, which we assume corresponds to an equilibrated system. As expected, the wider attractive potential (τ = 40 Å) leads to a greater cohesive energy. We have also carried out simulations for this larger τ system, where the amplitude Ψ_1_ of the attractive part is reduced to 60, giving rise to a substantially weaker cohesive energy in the clusters.

An obvious advantage with 2D simulations is that we can monitor coordinate snapshots via simple 2D plots, whereby separate clusters are easily discernible.

**Figure 4:**
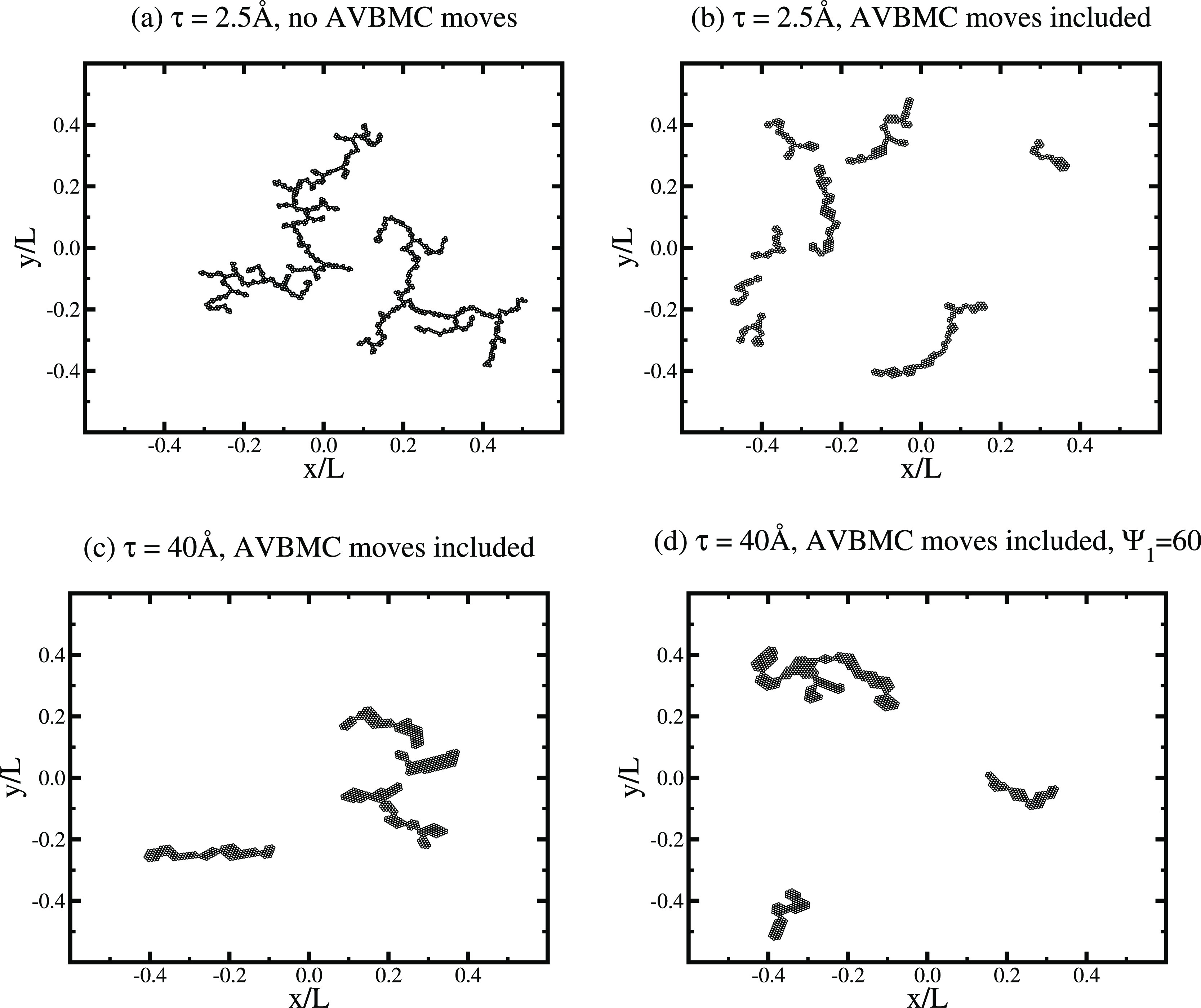
Snapshot coordinate plots of stable and metastable 2D systems. (a) τ = 2.5 Å, from simulations without AVBMC moves. (b) τ = 2.5 Å, from simulations with AVBMC moves. (c) τ = 40 Å, from simulation with AVBMC moves. (d) τ = 40 Å, from simulations with AVBMC moves. In this case, the amplitude of the attractive potential, Ψ_1_, was reduced to 60 (from the reference value of 70).

In the absence of AVBMC moves, we observe metastable clusters that are considerably larger, more branched, and with a thin “backbone”, Figure 4a. Overall, these clusters resemble branched polymers consisting of single strands of particles (at least for narrow attractive wells). The system is dilute, but it is tempting to conjecture the onset of gelation in more concentrated samples due to percolation. It should be noted that gels are indeed known to form in the experimental system (to which the reference potential was fitted to—see above) at least in three dimensions.^4^ This lends support to the notion that these metastable structures are likely more similar to what would be observed in an experimental scenario.

The metastable system described above emerges as a result of the large free energy barriers that prevent the formation of properly equilibrated structures. The latter are more likely to be observed in the presence of AVBMC moves—see Figure 4b–d. Here we see much less branching and the propensity to form polymeric forms with a thicker backbone, where the clusters display a mixture of double rods and what appear to be extended 2D lattice structures, which resemble elongated semicrystalline forms. The wider the attractive component in the PMF, the more compact will these equilibrium structures be—see Figure 4c,d, where we also note that reducing the magnitude of the attractive potential (Ψ_1_ from 70 to 60) seems to have little effect on the cluster morphology. The PMF with the narrower minimum generates mainly double-rod equilibrium structures, while the wider PMF system displays more compact configurations with a correspondingly lower energy. For tighter attractions, entropy dictates the formation of a greater number of smaller clusters, while for the wider attraction, there are more compact configurations with a low energy—compare Figure 4b–d.

**Figure 5:**
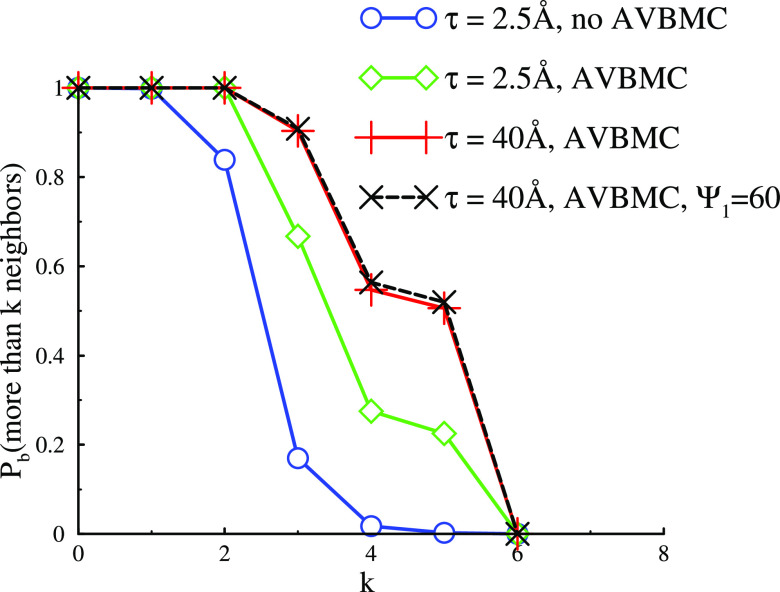
Accumulated neighbor number distributions for 2D clusters, that is, the probability *P*_b_(*k*) that a particle (monomer) has *k* or more cluster neighbors.

Figure 5 gives the bond probability distributions *P*_b_ for the different cases shown in Figure 4a–d. The metastable clusters shown in Figure 4a display mainly 2–3 nearest neighbors consistent with the single-stranded polymeric forms with the occasional branch points. Inclusion of the AVBMC means that the resultant more compact (semicrystalline) clusters display a larger number of nearest neighbors. For the narrow PMF (τ = 2.5 Å), we see the growth of a significant number of four and five nearest neighbors consistent with double strands and more extended 2D crystal forms. The probability of these increases for the wider PMF (τ = 40 Å), consistent with more clusters showing larger crystalline regions. Finally, we see that a drop of Ψ_1_ from 70 to 60 has virtually no impact on *P*_b_ at least not for the case we investigated with τ = 40 Å.

**Figure 6:**
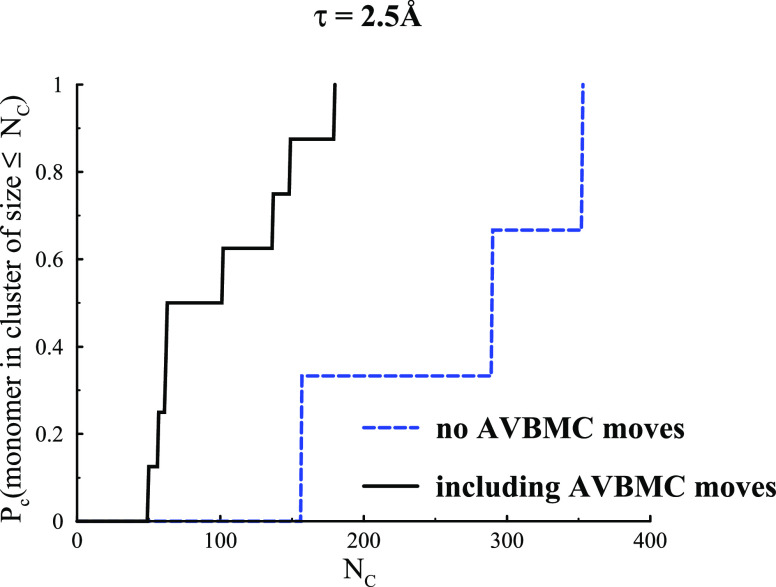
Integrated cluster size distributions, *P*_c_, for 2D systems with τ = 2.5 Å. The solid black line represents the metastable system that results in the absence of AVBMC moves, whereas the dashed blue line is for the fully equilibrated system.

In Figure 6, we show cluster size distributions, *P*_c_(*N*_c_), for the narrow (τ = 2.5 Å) 2D systems, as obtained both with and without AVBMC moves. The metastable clusters obtained without these moves show substantially larger cluster sizes, in comparison with the equilibrated clusters formed with AVBMC moves. It is interesting that the larger metastable clusters have less binding energy per particle, *U*/*N*, compared with the equilibrium clusters, as seen in Figure 3. For compact clusters, a larger number of smaller clusters will exhibit more surface, which can raise the average energy per particle if the surface tension is positive. The presence of finite clusters implies that cluster growth is impeded, most likely due to the repulsive interaction. The more open, branched clusters of the metastable case means that the repulsive contribution is weaker, allowing for more particles per cluster, but the overall average binding energy per particle is still low due to a reduced number of bonded neighbors.

### 3D Systems

**Figure 7:**
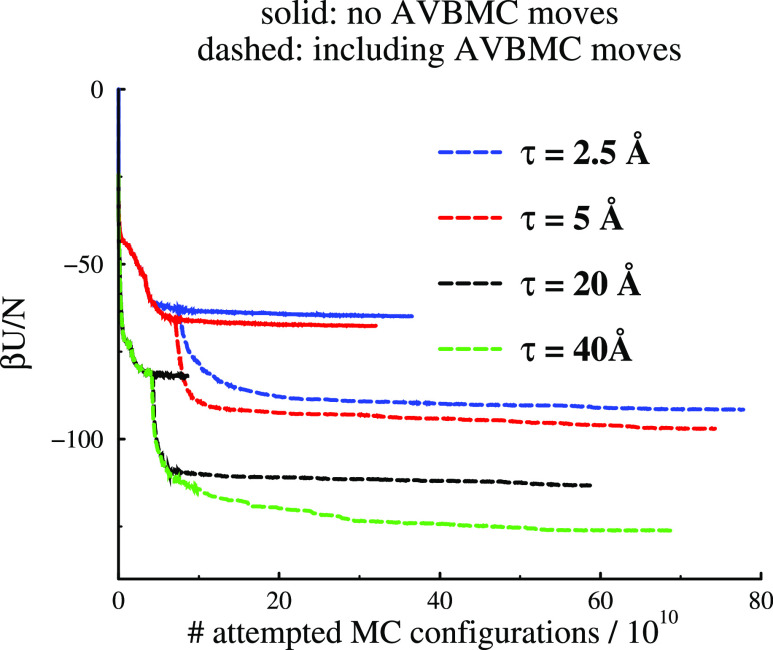
Energy convergences (3D). In most cases (except when τ = 40 Å), an initially semiequilibrated simulation (without AVBMC moves) was split along two different paths, one of which included AVBMC moves.

Here, we consider the simulation results for 3D systems. Energy convergence profiles are given in Figure 7. In most cases, we have utilized a “split”, whereby a simulation initially semiequilibrated without AVBMC moves is continued along two different paths: with or without AVBMC moves. An exception is the largest τ value considered (40 Å), where we only considered a path where the AVBMC moves were included. Again, in the absence of AVBMC moves, the systems become “trapped” in metastable states. We reiterate that these metastable states are likely to be a more realistic representation of experimental structures than the equilibrated states that result when AVBMC moves are utilized. We note that the metastable and stable energy levels of the two tightest PMFs, τ = 2.5 and 5 Å, are quite similar, with the latter being somewhat deeper. This similarity is also found structurally, and so, we will not display structural analyses of the τ = 2.5 Å simulations in the main paper, but we do provide structural snapshots in the Supporting Information.

We have chosen to illustrate structures via simulation snapshots of the largest cluster present for the given configurations. This makes it easier to scrutinize typical features. Corresponding snapshots of the entire systems are shown in the Supporting Information.

**Figure 8:**
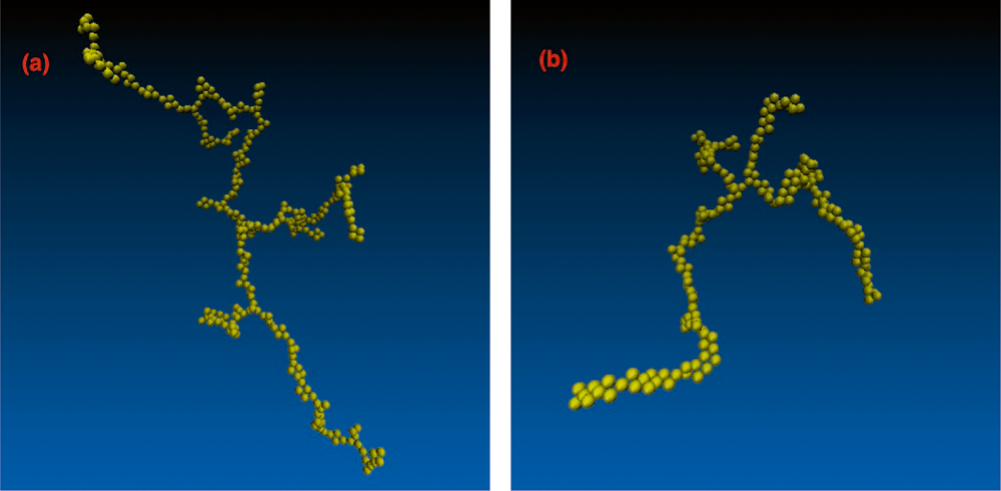
Illustrations of the largest clusters found from configurational snapshots of metastable 3D structures, as obtained with two different decay lengths of the Morse potential. Note that AVBMC moves were not implemented in these cases. These images were constructed using the VMD software.^33^ (a) τ = 5 Å. (b) τ = 20 Å.

**Figure 9:**
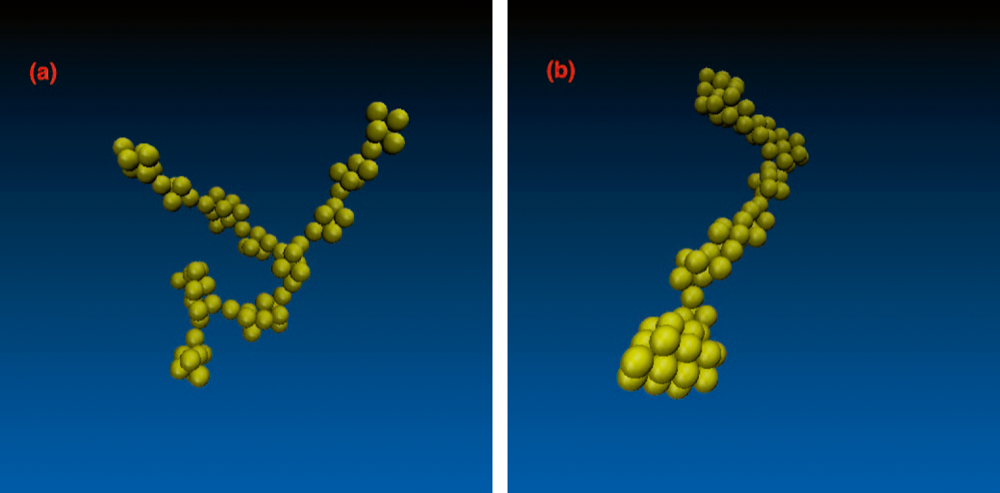
Illustrations of the largest clusters found from configurational snapshots of 3D structures as obtained with two different decay lengths of the Morse potential. AVBMC moves were implemented in these cases, that is, we expect these to represent typical clusters at complete equilibrium. These images were constructed using the VMD software.^33^ (a) τ = 5 Å. (b) τ = 20 Å.

Let us first focus on the clusters that are generated without AVBMC moves. Examples are provided in Figure 8. As in our 2D simulations, we observe rather extended and branched, polymer-like clusters with a relatively thin backbone, which become thicker for the wider attractive PMF. In fact, these clusters are rather similar to those observed in recent experiments by Haddadi et al. on PEO-grafted PS particles dispersed in aqueous solution.^30^ This supports our proposition that such metastable structures are a reasonable representation of those found in experimental samples. It should also be noted that the polymer-like structures that Haddadi et al. established at elevated temperatures remained intact upon cooling, which indicates that they are at least metastable. On the other hand, examples of more “equilibrated” structures, obtained via the inclusion of AVBMC moves, are shown in Figure 9. We again see that equilibrated structures are more compact polymeric forms with a thicker backbone and reduced branching. We also observe that the wider PMF produces more compact clusters still. For the two broadest PMFs considered, τ = 20 and 40 Å, there is even a representation of pyramid-shaped structures. These are reasonably similar to some of the energy-minimized structures found by Mossa et al.^34^ These compact clusters eventuate due to the greater number of particle configurations associated with significant cohesive interactions for the wide attractive well. With the narrow PMF, the system adopts instead higher entropy states by creating relatively longer (more flexible) branches with fewer bonded neighbors, though not to the same extent as in the metastable structures. The mutual repulsion between clusters may also play a role, where one would anticipate that more flexible clusters have the conformational freedom to better reduce the repulsion from neighboring clusters. Such a mechanism may also serve to stabilize the more extended metastable clusters.

**Figure 10:**
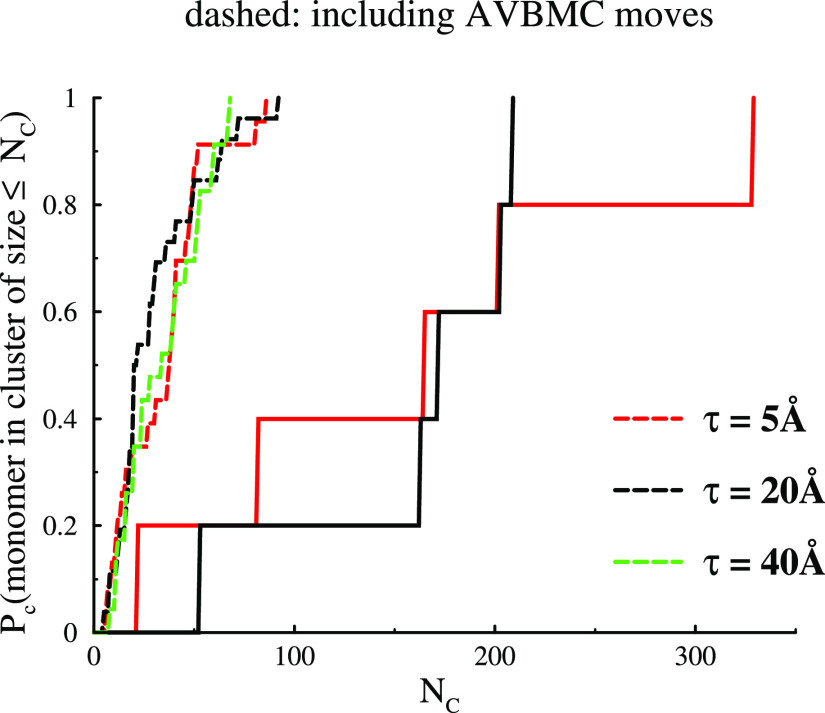
Integrated cluster size distributions, *P*_c_, for 3D systems with various widths of the Morse potential. Solid lines are for the metastable system that results in the absence of AVBMC moves, whereas dashed lines are for fully equilibrated systems.

The integrated cluster size distributions, *P*_c_, for metastable and fully equilibrated systems are shown in Figure 10. Let us first consider the metastable systems, represented by solid lines. One interesting observation is that the average cluster size distribution is essentially independent of the width of the attractive well of the PMF, at least within the examined region, τ = 5–20 Å. Given that very large clusters are present, and the fact that our system only contains 800 particles, we anticipate significant size effects. We can nevertheless again conclude that quite large clusters are formed in the absence of AVBMC moves. The remarkable observation we note here in these 3D simulations, in line with our findings in 2D systems, is that turning on AVBMC moves will lead to considerably smaller clusters. A plausible reason for this is that the long-ranged repulsion from a compact cluster prevents the cluster from growing beyond a certain critical size. For the more open-branched metastable clusters, the repulsive contribution is much weaker, which allows the clusters to reach fairly large sizes.

The accumulated neighbor number distribution is the probability *P*_b_(*k*) that a particle (monomer) has *k* or less cluster neighbors.

**Figure 11:**
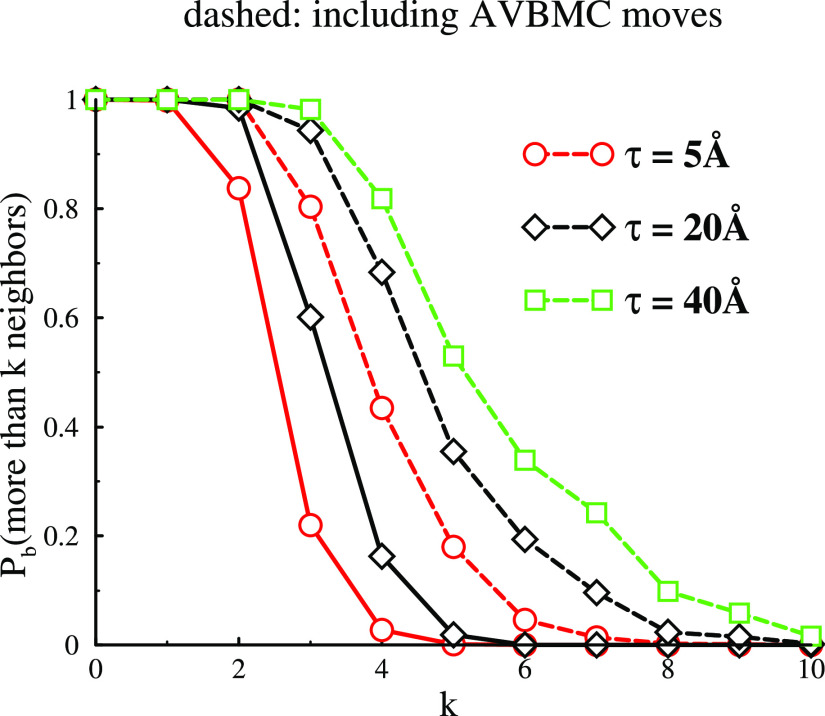
Structural properties of clusters formed for systems with two different widths of the attractive PMF regime (τ =5 and 20 Å).

The bond distributions, *P*_k_, for these systems are shown in Figure 11. First, we note that, for metastable and equilibrated clusters, a larger τ value generates structures where a particle on average has more neighbors. This is due to a higher entropic cost associated with confining many particles to narrow attractive wells. Another result that is quite clear from Figure 11 is that particles in the equilibrated clusters have a larger number of neighbors than in their metastable counterparts. This is in line with the observed increased cohesion (Figure 7).

## Conclusions

In this work, we have investigated the role played by the width of the PMF attractive wells on the cluster morphology in 2D and 3D systems. In addition to the short-ranged attractive wells, the PMFs also display a long-ranged electrostatic repulsion, giving rise to an energy barrier and a long-ranged repulsive tail. We have shown that the potential parameters that we have used are consistent with PMFs that can be realized in experimental systems of charged PS particles with attached (short) PEO chains. The PMFs used here have much deeper and narrower wells and stronger repulsive barriers, when compared with past theoretical reports on similar systems.^21,22,25^ For our choice of potentials, the ground-state (low temperature) structures consist of Bernal spirals^24^ in three dimensions rather than an extended crystalline phase. In two dimensions, the triangular lattice is only marginally more stable than a double rod.

We have demonstrated that, with the PMF parameter ranges used here, the width of the short-ranged well can have a significant effect on the cluster morphology, with more narrow wells promoting the formation of clusters in which a member has fewer nearest neighbors. If the cluster-swapping AVBMC moves are included in the simulations, the final clusters tend to be compact, but also quite small. In the absence of such moves, metastable conditions are obtained, with clusters akin to branched and quite extended polymers. The thickness of the backbone of these structures differs between systems with various values of the Morse potential decay parameter τ. Interestingly enough, these metastable clusters will on average contain a considerably larger number of particles than those found in the corresponding fully equilibrated systems. The findings mentioned above apply for 2D and 3D systems.

We argue, with some support by experimental observations,^30^ that the metastable structures are likely to be a better representation of typical experimental structures than their fully equilibrated counterparts. One could, in a somewhat hand-waving manner, argue that the AVBMC moves are likely to be “too efficient” in these systems, if the goal is predictions of experimental findings. However, in systems where barriers and minima are more modest, the final structures will not depend on the inclusion (or exclusion) of such moves.

We hope that the results of this work can guide experimental efforts to create specific supramolecular structures. We note that a potential problem in aqueous solutions is to keep the ionic strength low enough, so that a repulsive PMF is significant compared with the diameter of large particles. In principle, this can be addressed by reducing the size of the particles, but there is a lower bound for the particle size (about 100 nm), below which linear aggregates will not be detectable by confocal microscopy. This notwithstanding, our work has revealed a significant role played by the width in the attractive energy well for directing the most probable cluster morphology in a rather remarkable way.

## Supporting Information Available

Simulation snapshots of simulated 3D systems and comparisons with results obtained in a previous work on cluster formations where very different simulation methods were utilized^21^ (PDF)
