# Supplementary material for: Polymer-Like Self-Assembled Structures from Particles with Isotropic Interactions: Dependence upon the Range of the Attraction
Source: Langmuir. 2021 May 5;37(19):6052–61. doi: 10.1021/acs.langmuir.1c00719 (PMC8280719; doi:10.1021/acs.langmuir.1c00719)
Supplement: Supplementary file 1 — la1c00719_si_001.pdf [file la1c00719_si_001.pdf]

Supporting Information.

Polymer-like self-assembled structures from particles with isotropic interactions: dependence upon the range of the attraction.

6 pages, 4 Figures.

Sara Haddadi*, Hongduo Lu*, Marcus Bäcklund*, Clifford E. Woodward** and Jan Forsman**

**Theoretical Chemistry, P.O.Box 124, S-221 00 Lund, Sweden*

***University College, University of New South Wales, ADFA Canberra ACT 2600, Australia*

E-mail: jan.forsman@teokem.lu.se,phone:int+46462220381

Full system snapshots, in 3D

Here, we report coordinate snapshots, of 3D systems. Illustrating configurations typical to the metastable conditions, are provided in Figure S1. In a procedure analogous to our 2D studies, we have identified clusters, and ensured that these stays intact by removing the periodic boundary conditions.

Representative snapshots of fully equilibrated structures, established via the inclusion of AVBMC moves, are shown in Figure S2.

Reproducing some previously reported simulations results

Here, we simply demonstrate that we, as expected get the same results with our MC simulations as was previously reported by Sciortino *et al.*[2] Specifically, we shall focus on their "T route" simulation, at a particle volume fraction $\phi_c = 0.08$, and a reduced temperature kT $epsilon = 0.07$ (where ϵ id the energy unit in ref.[2] The system contained 2500 particles. We refer to ref.[2] for details.

In Figure S3, we report energy convergence for this system, as measured by the interaction energy per particle, scaled by the thermal energy, kT. The limiting value, about -16.4 can be compared with the $\phi_c = 0.08$ curve, at $kT/\epsilon = 0.07$, in Figure 7 of ref.[2] They chose to scale the interaction energy by the minimum value of the interaction potential, -0.52ϵ. At a reduced temperature of 0.07, our limiting plateau value for the interactiuon energy per particle, would then be scaled to about $-16.4 * 0.07/(-0.52) \approx 2.2$, which agrees well with the value found by Sciortino *et al.* (they report negative values, but we expect that to be a simple typo).

Switching focus to structure, our simulated particle-particle radial distribution function is provided in Figure S4. This curve ageees nicely with the corresponding one shown in

Figure 10 of ref.[2] (bottom graph, red curve, in their publication).

References

(1) Humphrey, W.; Dalke, A.; Schulten, K. *J. Molec. Graphics* **1996**, *14*, 33–38.

(2) Sciortino, F.; Tartaglia, P.; Zaccarelli, E. *The Journal of Physical Chemistry B* **2005**, *109*, 21942–21953, PMID: 16853852.

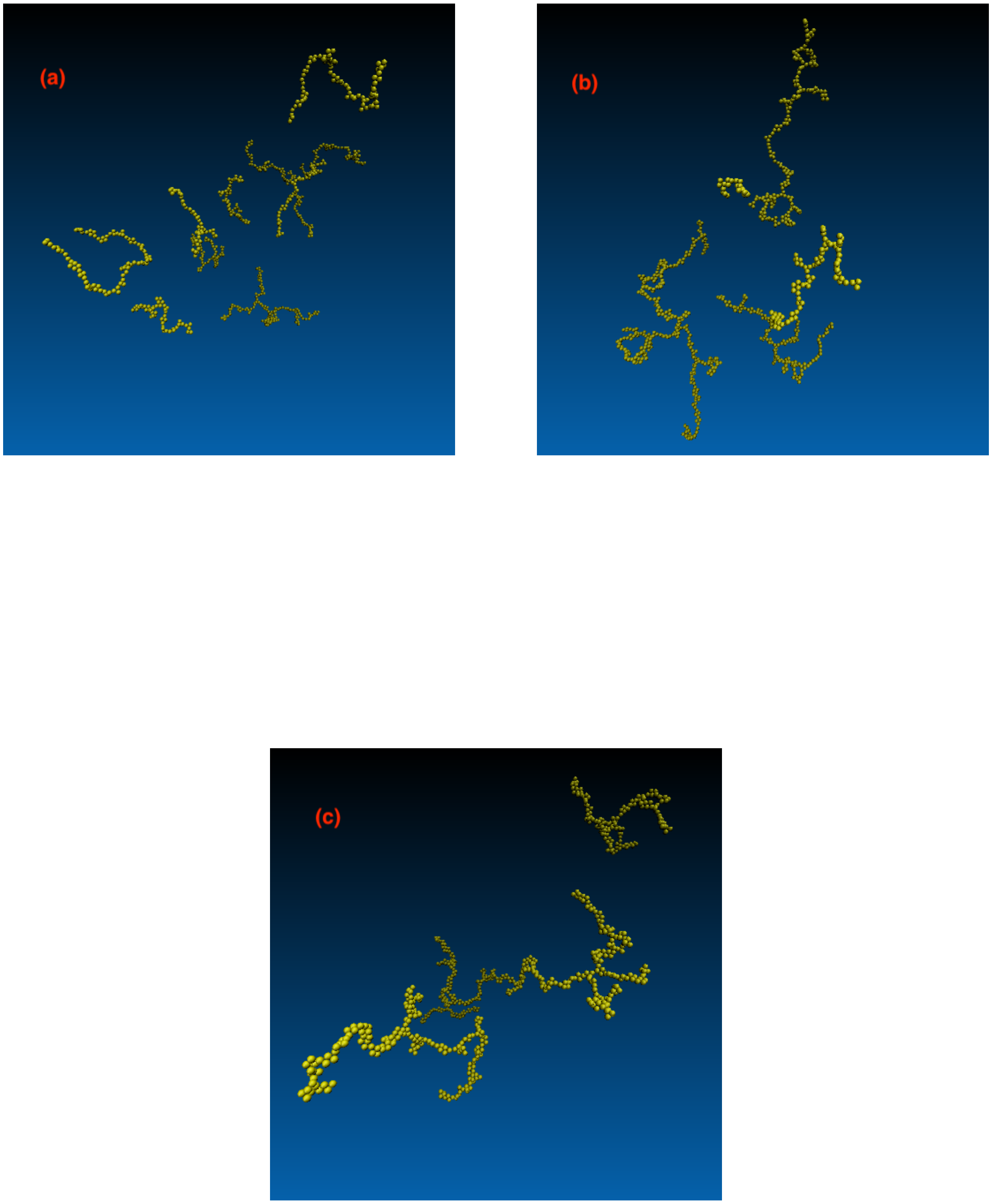

Figure S1: Configurational snapshots of metastable 3D structures, as obtained for different decay lengths of the Morse potential. AVBMC moves were *not* implemented in these cases. These images were constructed using the VMD software.[1]
(a) $\tau = 2.5$ Å.
(b) $\tau = 5$ Å.
(c) $\tau = 20$ Å.

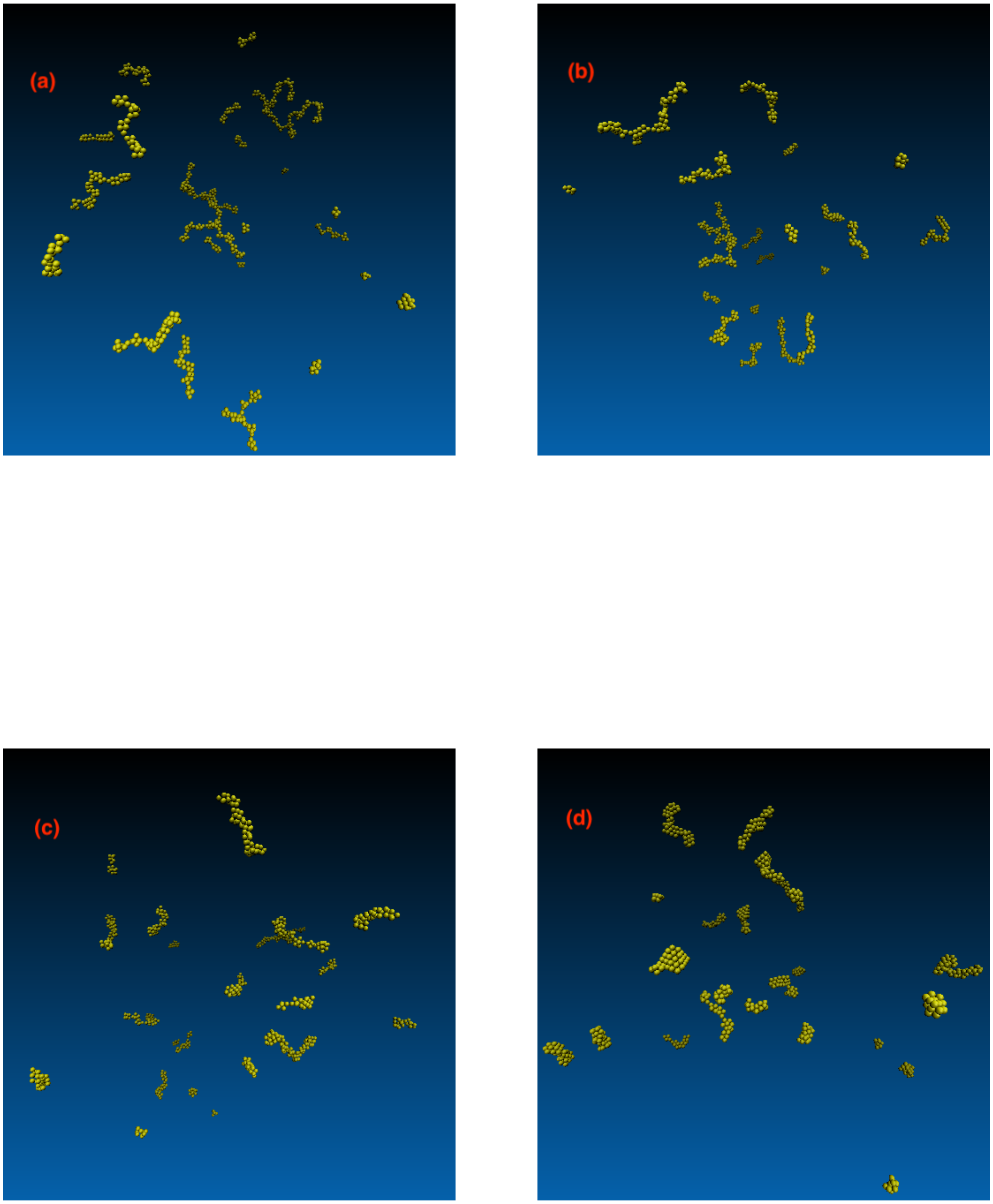

Figure S2: Configurational snapshots of 3D structures, as obtained for three different decay lengths of the Morse potential. AVBMC moves were included in all cases. These images were constructed using the VMD software.[1]
(a) $\tau = 2.5$ Å.
(b) $\tau = 5$ Å.
(c) $\tau = 20$ Å.
(d) $\tau = 40$ Å.

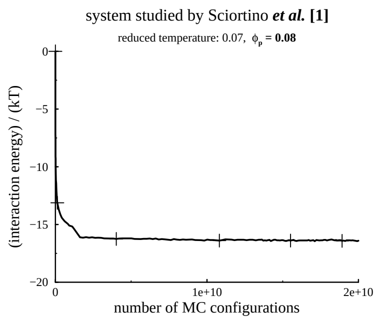

Figure S3: Interaction energy per particle, for one of the systems simulated by Sciortino *et al.*[2] - see main text.

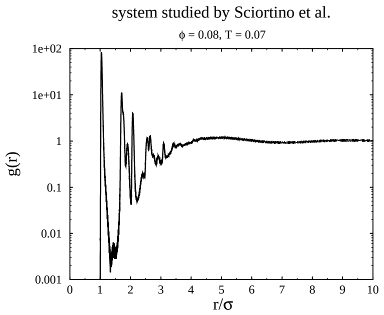

Figure S4: Particle-particle radial distribution function, $g(r)$, for one of the systems simulated by Sciortino *et al.*[2] - see main text.
